# The influences of PRG-1 on the expression of small RNAs and mRNAs

**DOI:** 10.1186/1471-2164-15-321

**Published:** 2014-04-30

**Authors:** Jia-Jia Wang, Dong-Ya Cui, Tengfei Xiao, Xubin Sun, Peng Zhang, Runsheng Chen, Shunmin He, Da-Wei Huang

**Affiliations:** School of Life Science, Hebei University, Hebei, 071002 People’s Republic of China; Key Laboratory of Zoological Systematics and Evolution, Institute of Zoology, Chinese Academy of Sciences, Beijing, 100101 People’s Republic of China; Laboratory of Bioinformatics and Noncoding RNA, Institute of Biophysics, Chinese Academy of Sciences, Beijing, 100101 People’s Republic of China; College of Life Science, Yuncheng University, Yuncheng, Shanxi 044000 People’s Republic of China

**Keywords:** *C. elegans*, miRNAs, 21U-RNAs

## Abstract

**Background:**

In metazoans, Piwi-related Argonaute proteins play important roles in maintaining germline integrity and fertility and have been linked to a class of germline-enriched small RNAs termed piRNAs. *Caenorhabditis elegans* encodes two Piwi family proteins called PRG-1 and PRG-2, and PRG-1 interacts with the *C. elegans* piRNAs (21U-RNAs). Previous studies found that mutation of *prg-1* causes a marked reduction in the expression of 21U-RNAs, temperature-sensitive defects in fertility and other phenotypic defects.

**Results:**

In this study, we wanted to systematically demonstrate the function of PRG-1 in the regulation of small RNAs and their targets. By analyzing small RNAs and mRNAs with and without a mutation in *prg-1* during *C. elegans* development, we demonstrated that (1) mutation of *prg-1* leads to a decrease in the expression of 21U-RNAs, and causes 35 ~ 40% of miRNAs to be down-regulated; (2) in *C. elegans*, approximately 3% (6% in L4) of protein-coding genes are differentially expressed after mutating *prg-1*, and 60 ~ 70% of these substantially altered protein-coding genes are up-regulated; (3) the target genes of the down-regulated miRNAs and the candidate target genes of the down-regulated 21U-RNAs are enriched in the up-regulated protein-coding genes; and (4) PRG-1 regulates protein-coding genes by down-regulating small RNAs (miRNAs and 21U-RNAs) that target genes that participate in the development of *C. elegans*.

**Conclusions:**

In *prg-1*-mutated *C. elegans*, the expression of miRNAs and 21U-RNAs was reduced, and the protein-coding targets, which were associated with the development of *C. elegans*, were up-regulated. This may be the mechanism underlying PRG-1 function.

**Electronic supplementary material:**

The online version of this article (doi:10.1186/1471-2164-15-321) contains supplementary material, which is available to authorized users.

## Background

Small non-coding RNAs, including microRNAs (miRNAs), Piwi-interacting RNAs (piRNAs), endogenous-siRNAs (endo-siRNAs) and others, play important roles in controlling gene expression. These small RNAs interact with different types of Argonaute proteins to form complexes, such as the RNA-induced silencing complex (RISC) [1–4]. These complexes recognize target genes via complementary base pairing and regulate the target genes’ expression. The *Caenorhabditis elegans* genome is currently known to encode 24 Argonaute proteins [5], which are divided into three subcategories based on homology and the small RNAs with which they interact: (1) PIWIs, which interact with the 21U-RNAs, or piRNAs; (2) Argonautes, two of which have been shown to interact with miRNAs, and two of which have been shown to interact with 26G-RNAs; and (3) the worm Argonautes (WAGOs), which interact with 22G-RNAs [6–16].

In *C. elegans*, two Piwi-related proteins, PRG-1 and PRG-2, have been identified. Loss of PRG-1 can cause germline defects and temperature-sensitive sterility [8, 17]. 21U-RNAs, the piRNAs of *C. elegans*, are precisely 21 nucleotides long which are shorter than piRNAs in flies and mammals, have a bias for uracil 5′ monophosphate and have a modified 3′ end that resists periodate-degradation [7, 8, 18–22]. 21U-RNAs are expressed in the germline. Their genomic loci disperse in two broad regions of chromosome IV [18], and their accumulation depends on the wild-type activity of PRG-1. Mutation of *prg-1* causes decreased expression of 21U-RNAs [8, 17].

*C. elegans* has complex interactions within its regulatory network. We would therefore expect, that some small RNAs other than 21U-RNAs, such as miRNAs and endo-siRNAs, are influenced directly or indirectly by PRG-1. To study whether PRG-1 can influence the expression of other small RNAs and regulate protein-coding genes via small RNAs, we extracted small RNAs from six developmental stages (embryo, L1, L2, L3, L4 and young adult) and mRNAs from four developmental stages (L1, L2, L3 and L4) of *prg-1*-mutant *C. elegans* for high-throughput sequencing. We obtained wild-type data of small RNAs and mRNAs from the corresponding stages from the NCBI database [8, 23]. We analyzed the wild-type and mutant *prg-1* data and demonstrated the function of PRG-1.

## Results

We extracted small RNAs from six developmental stages (embryo, L1, L2, L3, L4, and young adult) and mRNAs from four stages (L1, L2, L3 and L4) of *prg-1* mutants. High-throughput sequencing of small RNA samples produced 52,363,338 reads that mapped to the *C. elegans* genome. Sequencing of mRNA samples produced 48,257,011 mappable reads. The numbers of small RNA and mRNA reads there were generated at each stage are shown in Table 1.

**Table 1:** **Summary of the RNA-seq data**

Stage	Mappable reads	
	Small RNA	mRNA
Embryo	8,056,943	
L1	8,867,568	11,794,170
L2	8,632,646	11,842,726
L3	8,970,579	12,222,134
L4	8,941,933	12,397,981
Young adult	8,893,669	
Total reads	52,363,338	48,257,011

The reads of small RNAs for six stages (embryo, L1, L2, L3, L4, and young adult) and mRNAs for four stages (L1, L2, L3, and L4) in the *prg-1* mutant.

### The influence of PRG-1 on the composition of small RNAs

In *C. elegans*, small RNAs can be classified by their Argonaute-binding partners [24]. The expression of small RNAs, including miRNAs and 21U-RNAs, changes during development [25]. To test whether the composition of small RNAs in different stages are affected by PRG-1, we analyzed the composition of small RNAs with and without a *prg-1* mutation.

**Figure 1:**
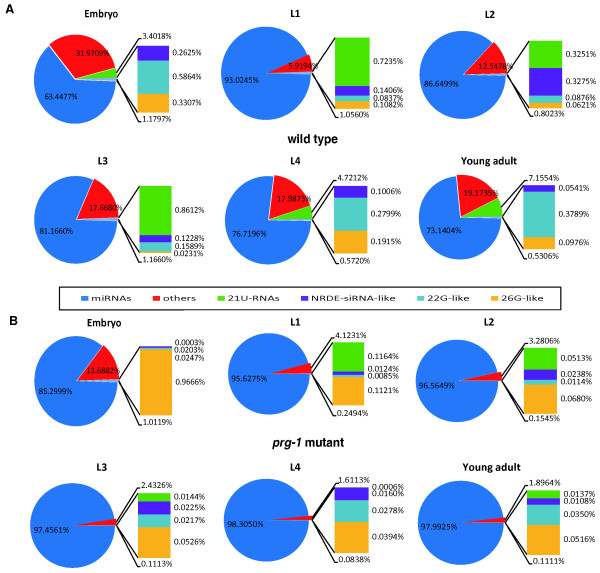
**The small RNA compositions of each stage in wild type and the** ***prg-1*** **mutant. (A)** The proportion of different types of small RNAs in six developmental stages of the wild type. **(B)** The proportion of different types small RNAs in six developmental stages of the *prg-1* mutant. “others” include rRNAs, tRNAs, snoRNAs and small RNA precursors and degradation products.

In wild-type *C. elegans* (Figure 1A), the percent of small RNAs that are 21U-RNAs gradually increased along with development, from 0.72% in L1 to 7.16% in young adults. However, the expression of 21U-RNAs decreased from L1 to L2; and the reasons behind this differential expression are described below. Like the 21U-RNAs, the proportion of 22G-like small RNAs, which are 22 nucleotides long and have a 5′-G, gradually increased from 0.08% in L1 to 0.37% in young adults. Along with the development, 21U-RNAs and 22G-like small RNAs Spearman’s rank correlation is 0.771. This indicated that 21U-RNAs and 22G-like small RNAs may have a positive correlation.

However, 21U-RNAs could barely be detected in *prg-1* mutants (Figure 1B), which was consistent with previous reports. Interestingly, 22G-like small RNAs also tended to increase throughout the growth period, but the rate of increase was smaller than in the wild type.

miRNAs were highly expressed and had absolute dominance in all developmental stages in the wild type and *prg-1* mutant. The proportion of small RNAs that were 26 nucleotides long and had a 5′-G, termed 26G-like small RNAs, decreased gradually during early development. Inversely, these RNAs exhibited a slight increase in late development. In wild type, the Spearman’s correlation of 22G-like small RNAs and 26G-like small RNAs is 0.486, so there is weaker correlation between 22G-like small RNAs and 26G-like small RNAs.

### Mutation of *prg-1* induced a decrease in 35 ~ 40% of miRNAs

miRNAs are well characterized in *C. elegans*[18, 24–29]. Mature miRNAs associate with the Argonautes proteins ALG-1 and ALG-2 [9]. However, it is not clear whether PRG-1 affects miRNAs. To explore whether mutation of *prg-1* affected miRNAs, we used the miRDeep2 program [30] to identify known miRNAs from all developmental stages. DEGseq [31] and GFOLD [32] were used to analyze miRNA expression at the same developmental stages in the presence or absence of a *prg-1* mutation, and the differential expression miRNAs were defined in ‘Methods’.

**Figure 2:**
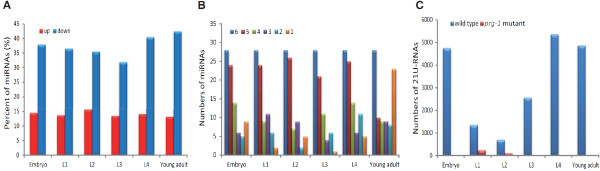
**Differential expression of miRNAs and 21U-RNAs in each stage with and without the** ***prg-1*** **mutation. (A)** The proportion of changed miRNAs in six stages. “up” indicates that the expression was up-regulated more than twofold after *prg-1* mutation; and “down” indicates that the expression was down-regulated more than twofold after *prg-1* mutation. **(B)** All miRNAs were classified by the number of times (1 ~ 6) that a miRNA was decreased in different stages. The time distribution of the down-regulated miRNAs is shown for each stage. For example, the red bar shows the number of the down-regulated miRNAs that were down-regulated in five stages. Most of the down-regulated miRNAs were down-regulated in five or six stages after mutation of *prg-1*. Thus, the decrease in the miRNA levels was independent of the developmental stage. **(C)** The numbers of expressed 21U-RNAs in six stages of wild type and *prg-1* mutant.

Approximately 50% of the known miRNAs exhibited changes in expression at the same stage when in the presence and absence of a *prg-1* mutation (Additional file 1: Table S1). At each stage, 35% ~ 40% of miRNAs showed a decrease (Figure 2A). The results indicated that PRG-1 affects miRNA expression. Many known miRNAs were decreased in the *prg-1* mutant. Therefore, PRG-1 plays an important role in regulating miRNA expression.

The read count of each miRNA differs between developmental stages; therefore it is reasonable to conclude that miRNA expression is stage-specific [25]. As mentioned above, some miRNAs exhibited a decrease in each stage after mutation of *prg-1*. The decreased miRNAs were stage-specific or general. To determine whether the effect of PRG-1 on miRNAs at different stages was specific, we performed further analysis of the decreased miRNAs in six stages. As exhibited in Figure 2B, most down-regulated miRNAs are shown to decrease in all developmental stages or in five stages. That is, in different developmental stages, the down-regulated miRNAs were almost identical. This result indicated that the influence of PRG-1 on miRNAs was independent of the developmental stage.

We selected the targets of the miRNAs which were down-regulated in all developmental stages and analyzed the functions of these targets by DAVID [33, 34]. We found that these targets were related with the growth and mitochondrion (Additional file 2: Figure S1), and the outcome was similar to GO analysis section below.

### 21U-RNAs are expressed at low levels in the *prg-1* mutant

21U-RNAs, another class of *C. elegans* non-coding small RNAs, specifically bind PRG-1 to form a complex that is important for germline function and fertility [8]. There have been reports that PRG-1 was required for the accumulation of 21U-RNAs [8]. In our data, known 21U-RNAs were identified based on the list of 21U-RNAs by Bagijn *et al*. [35], and novel 21U-RNAs (Additional file 3) were predicted using the criteria described by Bagijn *et al.*[35]. The levels of nearly all 21U-RNAs at each stage were dramatically reduced by the *prg-1* mutation. As presented in Figure 2C, 21U-RNAs are expressed at low levels in six stages after the mutation of *prg-1*. Especially in L3-L4, when 21U-RNAs accumulate, 21U-RNA expression could not be detected. This result demonstrated that PRG-1 affected the expression and accumulation of 21U-RNAs, which supported previously published results.

### The expression of miRNAs and 21U-RNAs during development

During *C. elegans* development, individual miRNAs have dynamic expression patterns [25]. The expression of 21U-RNAs also changes during development [8, 25]. To examine the entire range of expression of miRNAs and 21U-RNAs during *C. elegans* development in the wild type and the *prg-1* mutant, we parsed the expression changes of miRNAs and 21U-RNAs between adjacent developmental stages.

**Figure 3:**
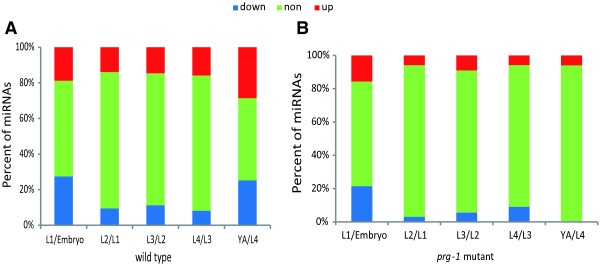
**Differential expression of miRNAs and 21U-RNAs between adjacent developmental stages.** The percent of differentially expressed miRNAs in the **(A)** wild type and **(B)** *prg-1* mutant between adjacent stages. “up” indicates that the expression (for example, L1/Embryo) was more than two-fold higher in later stages; "down" indicates that the expression (for example, L1/Embryo) was more than two fold lower in later stages; and “non” indicates that the expression between adjacent stages was up-regulated and down-regulated by less than twofold.

As observed in Figure 3A and B, ~68% of known miRNAs did not show a change between adjacent development stages, approximately 205 miRNAs were expressed in both adjacent periods (Additional file 1: Table S2). Moreover, in the *prg-1* mutant, expression of ~87% of the miRNAs did not differ significantly during development. The adjacent stages expressed approximately 214 miRNAs (Additional file 1: Table S2).

The expression of 21U-RNAs increased during development in wild-type *C. elegans* (Figure 2C). However, some 21U-RNAs were not expressed from the L4 to young adult stage. This phenomenon suggested that some 21U-RNAs were not needed in the mature organism; therefore, 21U-RNAs ceased being expressed and were gradually degraded. In the *prg-1* mutant, few 21U-RNAs could be detected, and those that were detected were low.

The general trend of 21U-RNAs is that the types gradually increase and that their expression increases during development in the wild type.

### Of the 3% of protein-coding genes that were substantially altered, approximatley 60 ~ 70% were up-regulated

**Figure 4:**
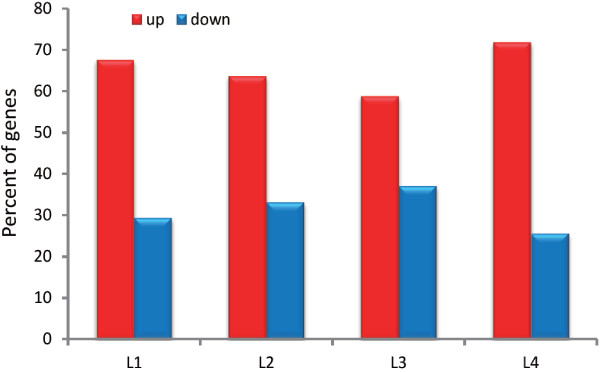
**Differentially expressed protein-coding genes after** ***prg-1*** **mutation.** The proportion of up- and down-regulated protein-coding genes that were significantly changed (P < 0.05, q < 0.01 of Storey) after *prg-1* mutation is shown.

PRG-1 influences the *C. elegans* reproductive phenotype, and phenotypic changes are directly dependent on the expression of protein-coding genes. Therefore, we expected to find some changes in gene expression in the *prg-1* mutant. To study whether PRG-1 affected the expression of protein-coding genes, the mRNA expression in four stages (L1-L4) with and without a mutation in *prg-1* were analyzed. We found that in L1-L4, 3.62%, 3.58%, 3.53% and 6.00%, respectively, of protein-coding genes were differentially expressed (Additional file 1: Table S3). Approximately 60 ~ 70% of the differentially expressed genes were up-regulated at each stage (Figure 4).

### The target genes of down-regulated small RNAs were up-regulated

miRNAs are small RNAs that regulate protein-coding genes, and 21U-RNAs are reported to participate in regulating protein-coding genes [35]. In our study, miRNA and 21U-RNA expression was reduced after mutation of *prg-1*, and we speculated that the reduction in small RNA expression led to the elevation of expression of protein-coding genes. To verify whether the target genes of the down-regulated small RNAs were up-regulated, we analyzed the target genes of the down-regulated miRNAs and 21U-RNAs in four stages.

**Figure 5:**
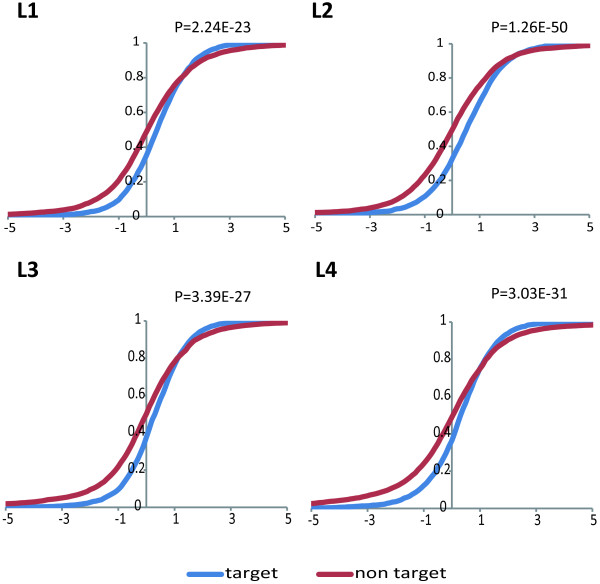
**The cumulative distribution of the targets of the down-regulated miRNAs and 21U-RNAs.** The cumulative distribution of the target and non-target genes of the down-regulated miRNAs and 21U-RNAs in L1-L4 indicated that the expression of the target genes was increased compared with that of non-target genes after *prg-1* mutation.

The results (Figure 5 includes P values from T-tests) indicated that the target genes of the down-regulated miRNAs and 21U-RNAs had higher expression in the same stages after mutation of *prg-1*. These findings suggest that PRG-1-dependent small RNAs affect the expression of protein-coding genes.

### Significantly up-regulated genes were enriched with the substantially altered target genes of the down-regulated miRNA and 21U-RNAs

The *prg-1* mutation led to a significant increase in the expression of some protein-coding genes. Meanwhile, the target genes of the down-regulated miRNA and 21U-RNAs were up-regulated. Therefore, we explored whether the up-regulated genes were induced by the down-regulated miRNAs and 21U-RNAs. The enrichment of the differentially expressed target genes that were regulated by the down-regulated miRNAs and 21U-RNAs within the substantially up-regulated protein-coding genes was calculated.

**Figure 6:**
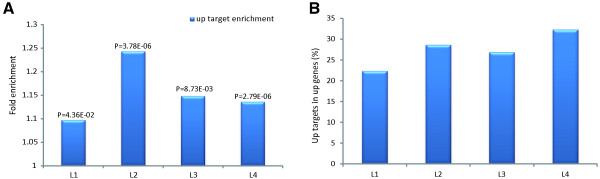
**The substantially increased protein-coding genes were enriched with targets of the down-regulated miRNAs and 21U-RNAs. (A)** The up-regulated genes were enriched with the differentially expressed target genes of the down-regulated miRNAs and 21U-RNAs at the corresponding stages. P values were calculated using Fisher’s exact test. **(B)** The percentage of up-regulated targets among the up-regulated protein-coding genes in the corresponding stages.

As seen in Figure 6A, the substantially up-regulated protein-coding genes were enriched with the differentially expressed target genes of the down-regulated miRNAs and 21U-RNAs (P values from Fisher’s exact test are shown in the figure). Of the up-regulated protein-coding genes, ~30% (Figure 6B) were up-regulated target genes. Namely, the *prg-1* mutation increased gene expression, and the down-regulation of miRNAs and 21U-RNAs was the cause of the increased gene expression in 1/3 of the substantially up-regulated protein-coding genes.

### PRG-1-dependent small RNAs participated in *C. elegans* development

Mutation of *prg-1* can affect small RNAs, thereby influencing the expression of their target genes. Alterations in the expression of target genes may change certain biological processes. To study how *prg-1* affects the *C. elegans* biological phenotype, GO analysis [33, 34] was performed for the up-regulated genes that were targets of the down-regulated miRNAs and 21U-RNAs in each stage.

**Figure 7:**
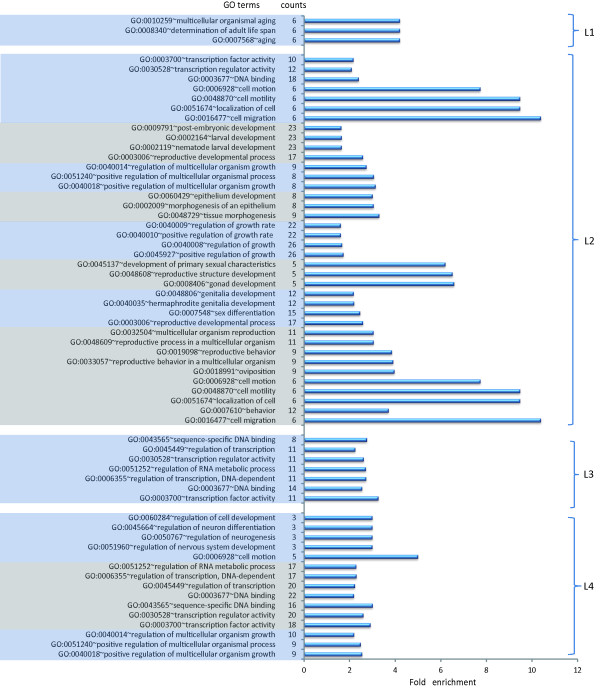
**GO analysis for the up-regulated targets of the down-regulated miRNAs and 21U-RNAs.** GO analysis was performed for the up-regulated target genes (P < 0.05, q < 0.01 of Storey) of the down-regulated miRNAs and 21U-RNAs. The GO terms were selected from clusters with a cluster enrichment score greater than 1 and P < 0.05. Counts indicate the genes in GO terms. Each color indicates one cluster.

The results (Figure 7) indicated that these target genes from different stages were all enriched in the biological processes related to growth and development. For example, some target genes were enriched in ‘determination of adult life span’ in L1. Target genes were also enriched in ‘regulation of multicellular organism growth’ in L2 and L4, as well as in ‘larval development’ in the L2 stages. Together, these findings illustrated that PRG-1-related protein-coding genes were involved in *C. elegans* development. If PRG-1 was expressed at normal levels, miRNAs and 21U-RNAs would be expressed normally, and their target genes would maintain normal levels of expression. Under these conditions, *C. elegans* would develop into a typical mature individual. In L3 and L4, these up-regulated genes were also enriched in the biological processes of transcription and RNA metabolism. In L3 in *C. elegans*, a large number of small RNAs, such as 21U-RNAs and 22G-RNAs, are produced, and the regulatory functions of small RNAs are reinforced. Both of these behaviors require a large number of transcriptional events. Therefore, these requirements explain the enrichment of genes involved in transcription- and RNA metabolism-related biological processes in the L3 and L4 stages.

### Three cases of verified, decreased miRNA targets

**Figure 8:**
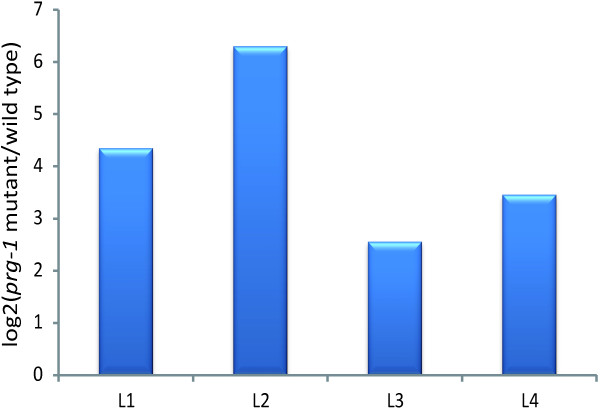
**Fold change of a target, K06A9.1, in L1-L4.** miR-63-3p, miR-66-5p, miR-87-3p, miR-233-3p and miR-234-3p were decreased after mutation of *prg-1*. Their target, K06A9.1, showed an increase of more than twofold of that in L1-L4. *prg-1* mutant and wild type indicate the expression of K06A9.1 in the *prg-1* mutant and wild type, respectively.

We systematically analyzed the function of the predicted target genes of the down-regulated miRNAs via DAVID and found that these target genes were related to development. Then, we downloaded the verified miRNA targets from the miRTarBase website [36] and selected three examples of target genes of the decreased miRNAs for analysis. miR-63-3p, miR-66-5p, miR-87-3p, miR-233-3p and miR-234-3p were decreased in the L1-L4 stages in the *prg-1* mutant. At the same time, their target, K06A9.1, displayed more than two-fold up-regulation (Figure 8). GO identified K06A9.1 as an intrinsic component of the membrane (GO:0031224). miR-60-3p was decreased in L1, L3 and L4, and its target, K12H4.4, had a greater than two-fold increase in the corresponding stages. miR-80-3p was down-regulated in L2-L4, and the expression of its target, B0361.9, increased more than two-fold after the mutation of *prg-1*. K12H4.4 and B0361.9 are both implicated in the development of *C. elegans*.

## Discussion

### Composition of other small RNAs

Our results included small RNAs that were not the focus of the current study, such as rRNAs (ribosomal RNAs), tRNAs (transfer RNAs), and snoRNAs (small nucleolar RNAs), as well as small RNA precursors and degradation products. In Figure 1, these RNAs (termed “other”) in the *prg-1* mutant had remarkable decreases in all stages relative to the wild-type *C. elegans*. It is likely that our results included types of small RNAs that have not yet been recognized, and these unknown small RNAs may have disappeared in the *prg-1* mutant. Alternately, the difference may be explained by the processes for preparing the small RNAs and sequencing.

### 22G-like and 26G-like small RNAs

In wild-type *C. elegans* (Figure 1A), the proportion of 21U-RNAs was reduced between the L1 and L2 stages. This phenomenon could be explained by an absence of expression of novel 21U-RNAs and prioritizing the degradation of 21U-RNAs during that period or to the fact that the rate at which 21U-RNAs were generated was less than the speed of their degradation. Either explanation would lead to an overall reduction in 21U-RNAs.

The present report has demonstrated that 21U-RNA-mediated silencing in the *C. elegans* germline results in secondary siRNA-dependent silencing of a ‘piRNA sensor’ [35]. Thus, the 21U-RNA-mediated silencing pathway completes the supervisory function through a secondary siRNA, known as 22G-RNAs which are RNA-dependent RNA polymerase (RdRP)-generated RNAs [35, 37]. 22G-RNAs are predominantly 22 nt in length and contain a 5′-G that is triphosphorylated [38]. As our data indicate, we only obtained 5′-monophosphate small RNAs; and no 22G-RNAs were detected, so we defined these 22 nt small RNA which selected by the targets of 22G-RNAs as 22G-like small RNA. 26G-like small RNAs also defined in a similar method. However, as the wild-type results show in Figure 1A, the expression of 22G-like small RNAs gradually increased during development, as did the expression of 21U-RNAs. Therefore, it was probable that some 5′-monophosphate 22G-RNAs were present and participated in downstream regulation of the 21U-RNA-mediated pathway.

There are two distinct classes of 26G-RNAs in *C. elegans*. One class is enriched in oocytes and embryos and associates with ERGO-1 [12, 15, 39]. The other class associates with ALG-3 and ALG-4 during spermatogenesis[10, 15]. Both classes are thought to function by triggering the formation of 22G-RNAs and subsequent silencing of target mRNAs [24, 40]. In wild-type *C. elegans*, the expression of 26G-like small RNAs first decreased and then increased as development progresses. A similar expression pattern was also observed in the *prg-1* mutant; however, the expression was lower than that of the wild type. Interestingly, in the *prg-1* mutant, the expression of 22G-like small RNAs increased during development (Figure 1B). If the *prg-1* mutation is present, the 21U-RNA-mediated pathway should be blocked, and the expression of 22G-like small RNAs, which are expressed downstream of this pathway, should decrease. However, the *prg-1* mutation did not affect the 26G-RNA-mediated pathway because the expression pattern of the 26G-RNAs did not change. The downstream 22G-like small RNAs were expressed normally and increased during development in the *prg-1* mutant.

### PRG-1-dependent, down-regulated miRNAs and 21U-RNAs are responsible for 1/3 of the expression of the substantially up-regulated genes

Approximately 60 ~ 70% of the significantly altered genes exhibited up-regulation after the mutation of *prg-1*. Of these, 1/3 were induced by the down-regulated miRNAs and 21U-RNAs. The remaining substantially up-regulated protein-coding genes might be directly regulated by the PRG-1 protein; the target genes of the small RNAs could regulate the other mRNAs. The internal control network of *C. elegans* is complex and cannot be fully explained by the *prg-1* pathway alone.

## Conclusions

We analyzed small RNAs (from embryo, L1, L2, L3, L4, and young adult) and mRNAs (from L1, L2, L3, and L4) in a *prg-1* mutant using high-throughput sequencing. By analyzing wild-type small RNAs and mRNAs of the corresponding stages found in the NCBI database, we found decreased miRNA and 21U-RNA levels in six stages after mutation of *prg-1*. In the *prg-1* mutant, approximately 3% of the protein-coding genes showed differential expression, of which approximately 60 ~ 70% exhibited up-regulation. Approximately 1/3 of the substantially up-regulated protein-coding genes were target genes of the down-regulated miRNAs and 21U-RNAs.

## Methods

### Small RNA and mRNA preparation and high-throughput sequencing

The nematode strain used in this study was the *C. elegans prg-1* mutant (*wm161*). Worms were cultured with the bacterial strain OP50 on nematode growth medium. All strains were grown at 20°C.

As described by Deng *et al*. [41], we collected small RNAs (embryo, L1, L2, L3, L4, and young adult) and mRNAs (L1-L4) at different stages. Total RNA was extracted from each of the six different developmental stages using the Trizol protocol. Small RNAs were size-selected by gel electrophoresis, and we used poly(A) to extract L1-L4 mRNA. Then, we submitted small RNAs from six stages and mRNA from four stages for high-throughput sequencing.

### Small RNA classification

First, we removed the sequences with lengths <18 nt and removed the simple repeat sequences that had a single-base content greater than 85%. Then, small RNAs were mapped to the *C. elegans* genome (ce10), with allowances for 0 mismatches using the software Bowtie.

The known miRNAs were selected from the perfectly mapped small RNAs using miRDeep2 [30]. 21U-RNAs were assessed using perfect matching to known 21U-RNAs from the perfectly matched small RNAs. We predicted the novel 21U-RNAs as Bagijn *et al*. described [35]. We also selected type 2 21U-RNAs [42].

The small RNAs that remained after we removed the miRNAs and 21U-RNAs were classified as described by Zhang *et al*. [38]. Briefly, published targets of different class siRNAs were parsed. The target genes of WAGO-1-12-associated 22G-RNAs [11] and of CSR-1-associated 22G-RNAs [14] were used to select 22G-like small RNAs. The target genes of ERGO-1 [12] and of ALG-3/4-associated 26G-RNAs [10, 15] were used to select the 26G-like small RNAs. The NRDE-like small RNAs were those that were identical to the NRDE-3-associated siRNAs [38]. The remaining perfectly mapped small RNAs were called ‘others’.

We used DEGseq [31] and GFOLD [32] to analyze miRNAs expression. We chose miRNAs which had more than two-fold difference in expression (P < 0.001, q < 0.01 of Storey) from DEGseq, and miRNAs which had more than two-fold difference in expression (GFOLD score > 0 for up-regulation and GFOLD score < 0 for down-regulation) from GFOLD outcomes. Then we obtained the intersection of up-regulated miRNAs and down-regulated miRNAs for each stage from the chosen miRNAs, respectively. 21U-RNAs (known 21U-RNAs) reads were normalized to the total known miRNA reads. Almost all of 21U-RNAs could not be detected after *prg-1* mutation, so we considered these 21U-RNAs were down-regulated. We considered 21U-RNAs expression if the expression is greater than 1 after normalization.

### mRNA data analyses and target gene screening

We used eight sets of mRNA data. The wild-type mRNAs (L1-L4) were downloaded from the NCBI database (GSE22410) [23]. The other four sets consisted of our sequencing data. Tophat and Cufflinks [43] were used to assemble the wild-type and *prg-1*-mutant mRNAs. Cuffdiff and DEGseq [31] were used to calculate the differential expression of protein-coding genes with and without the *prg-1* mutation, and we selected genes which had more than two-fold difference in expression (P < 0.05, q < 0.01 of Storey) from DEGseq outcomes. The intersection of genes which we selected from DEGseq outcomes and genes which had more than two-fold difference in expression (P < 0.05) from Cuffdiff outcomes was defined as differentially expressed genes. The following analyses were based on P < 0.05 and q < 0.01 of Storey.

The list of miRNA target genes was downloaded from microRNA.org. The target genes of the down-regulated miRNAs were chosen when five or more miRNAs had the same target genes. We predicted 21U-RNA candidate target genes in the *C. elegans* mRNAs and allowed for up to three mismatches [35].

### Gene ontology analyses

The up-regulated genes with P < 0.05 (q < 0.01 of Storey) were selected from the target genes of the down-regulated miRNAs and 21U-RNAs. The selected genes were input into DAVID [33, 34], which sorted these genes into functionally related clusters. The clusters with an enrichment score greater than 1 were chosen. The P < 0.05 GO terms were selected from the high enrichment score clusters.

### Availability of supporting data

The small RNAs and mRNAs data of wild type used in this study were downloaded from NCBI gene Expression Omnibus (http://www.ncbi.nlm.nih.gov/geo/) under accession number: GSE22410 and GSE11738; and small RNAs and mRNAs data of the *prg-1* mutant were deposited in the Gene Expression Omnibus with the following accession number: GSE56274. The additional files and information are available at the website: http://www.regulatoryrna.org/pub/cel_smallRNA/index.html.

## Electronic supplementary material

Additional file 1: Table S1: – miRNA expression after *prg-1* mutation. **Table S2** – miRNA expression during development. **Table S3** – Expression of protein-coding genes after *prg-1* mutation (P < 0.05, q < 0.01 of Storey). (PDF 122 KB)

Additional file 2: Figure S1: GO analysis for the targets of down-regulated miRNAs in all developmental stages. We selected the targets of the miRNAs which are down-regulated in all developmental stages and analyzed the functions of these targets by DAVID. Counts indicated the genes in GO terms. (PDF 563 KB)

Additional file 3: **The novel 21U-RNAs.** (XLSX 14 KB)
