# Supplementary material for: The influences of PRG-1 on the expression of small RNAs and mRNAs
Source: BMC Genomics. 2014 Apr 30;15(1):321. doi: 10.1186/1471-2164-15-321 (PMC4035053; doi:10.1186/1471-2164-15-321)
Supplement: Supplementary file 1 — Additional file 1: Table S1: – miRNA expression after prg-1 mutation. Table S2 – miRNA expression during development. Table S3 – Expression of protein-coding genes after prg-1 mutation (P < 0.05, q < 0.01 of Storey). (PDF 122 KB) [file 12864_2014_6021_MOESM1_ESM.pdf]

Table S1 miRNA expression after *prg-1* mutation

	Embryo	L1	L2	L3	L4	Young adult
**Expressed miRNAs**	227	219	217	223	220	205
**up**	33	30	34	30	31	27
**down**	86	80	77	71	89	87
**up(%)**	14.54	13.70	15.67	13.45	14.09	13.17
**down(%)**	37.88	36.53	35.48	31.84	40.45	42.44
**changed (%)**	52.42	50.23	51.15	45.29	54.54	55.61

Table S2 miRNA expression during development

	**wild type**					***prg-1* mutant**				
	**L1/Embryo**	**L2/L1**	**L3/L2**	**L4/L3**	**YA/L4**	**L1/Embryo**	**L2/L1**	**L3/L2**	**L4/L3**	**YA/L4**
**Expressed miRNAs**	208	201	205	208	206	224	223	212	209	203
**up**	39	28	30	33	59	35	13	19	12	12
**non**	112	154	152	158	95	141	203	181	178	190
**down**	57	19	23	17	52	48	7	12	19	1
**up(%)**	18.75	13.93	14.63	15.87	28.64	15.63	5.83	8.96	5.74	5.91
**non(%)**	53.85	76.62	74.15	75.96	46.12	62.94	91.03	85.38	85.17	93.60
**down(%)**	27.40	9.45	11.22	8.17	25.24	21.43	3.14	5.66	9.09	0.49

Table S3 Expression of protein-coding genes after *prg-1* mutation (P<0.05, q<0.01)

	**L1**	**L2**	**L3**	**L4**
**total genes**	16111	15998	15976	16220
**changed genes**	584	573	564	974
**up**	407	377	346	718
**down**	177	196	218	256
**changed genes(%)**	3.62	3.58	3.53	6.00
**up(%)**	69.69	65.79	61.35	73.72
**down(%)**	30.31	34.21	38.65	26.28
