# Supplementary figures and images for: The influences of PRG-1 on the expression of small RNAs and mRNAs
Source: BMC Genomics. 2014 Apr 30;15(1):321. doi: 10.1186/1471-2164-15-321 (PMC4035053; doi:10.1186/1471-2164-15-321)

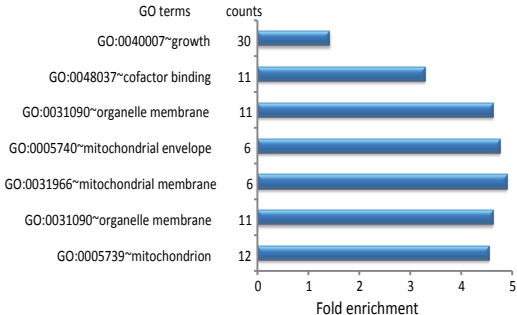

Supplement: Supplementary file 2 — Additional file 2: Figure S1: GO analysis for the targets of down-regulated miRNAs in all developmental stages. We selected the targets of the miRNAs which are down-regulated in all developmental stages and analyzed the functions of these targets by DAVID. Counts indicated the genes in GO terms. (PDF 563 KB) [file 12864_2014_6021_MOESM2_ESM.pdf]
